# High genetic homogeneity points to a single introduction event responsible for invasion of *Cotton leaf curl Multan virus* and its associated betasatellite into China

**DOI:** 10.1186/s12985-015-0397-y

**Published:** 2015-10-07

**Authors:** Zhenguo Du, Yafei Tang, Zifu He, Xiaoman She

**Affiliations:** Plant Protection Research Institute, Guangdong Academy of Agricultural Sciences, Guangzhou, 510640 China; Guangdong Provincial Key Laboratory of High Technology for Plant Protection, Guangzhou, 510640 China

**Keywords:** CLCuMuV, Invasion, Single introduction, Begomovirus, Whitefly Transmitted Geminivirus

## Abstract

**Background:**

*Cotton leaf curl Multan virus* (CLCuMuV) is a Whitefly Transmitted Geminivirus (WTG) endemic to the India subcontinent and is notorious as a causal agent of cotton leaf curl disease (CLCuD), a major constraint to cotton production in south Asia. We found CLCuMuV infecting *Hibiscus rosa-sinensis* in Guangzhou, China in 2006. The spread and evolution of the invading CLCuMuV were monitored in the following nine years.

**Findings:**

CLCuMuV spread rapidly in the last nine years and became established in Southern China. It infects at least five malvaceous plant species, *H. rosa-sinensis, H. esculentus*, *Malvaiscus arboreus, Gossypium hirsutum* and *H. cannabinus*. Complete nucleotide sequences of 34 geographically and/or temporally distinct CLCuMuV isolates were determined and analyzed together with six other publicly available genomes of CLCuMuV occurring in China. The 40 CLCuMuV isolates were found to share > 99 % nucleotide sequence identity with each other. In all cases tested, the CLCuMuVs were associated with a CLCuMuB. The 36 CLCuMuBs (30 sequenced by us) shared > 98 % nucleotide sequence identity.

**Conclusion:**

The high genetic homogeneity of CLCuMuV and CLCuMuB in China suggests the establishment of them from a single founder event.

**Electronic supplementary material:**

The online version of this article (doi:10.1186/s12985-015-0397-y) contains supplementary material, which is available to authorized users.

Viruses constitute an important group of pathogens that have been spread through the globalization of trade and travel. Viral introduction is responsible for a number of emerging infectious diseases (EIDs) that directly affect human living or affect human well-being indirectly by damaging poultry, livestock and causing population decline or even species extinction of wild animals [5, 9]. Viral introduction also poses a serious threat to plant cultivation. It is estimated that virus introductions account for about half of plant EIDs that cause huge economic losses and threaten food safety [2].

*Geminiviridae*, a family of single-stranded DNA (ssDNA) virus with characteristic twin icosahedral particles, has emerged as one of the most important groups of plant pathogens [13]. The earliest record describing infection of a geminivirus dates back to 752 AD [20]. However, recent phylogenic studies showed that most geminiviruses are grouped according to their geographical origins [15]. This indicated that extensive long-distance movement of geminiviruses did not occur for centuries. The situation began to change in the end of the last century due to many anthropogenic activities that have favored virus dispersal [21]. This is especially obvious for Whitefly Transmitted Geminiviruses (WTGs), which usually infect only dicotyledonous plants and are taxonomically classified into the genus *Begomovirus*. Geographical expansion of some WTGs has resulted in many EIDs that severely threaten crops such as cassava, cotton and tomato, causing famine and the death of thousands of people in some underdeveloped countries [10, 16, 17, 19]. Besides threatening their host plants directly, introduced WTGs provide novel genetic resources to local viruses for recombination, which has the potential to create viruses with novel pathogenic properties that may cause new disease epidemics [11].

Cotton leaf curl disease (CLCuD) is one of the most notorious plant diseases caused by WTGs. It represents a major limiting factor for cotton production in many regions of South Asia [19, 26]. *Cotton leaf curl Multan virus* (CLCuMuV) associated with a betasatellite called Cotton leaf curl Multan betasatellite (CLCuMuB) is one of the five begomoviruses responsible for an outbreak of CLCuD, which devastated the cotton industry of South Asia during 1991–2001 [19]. During the late 1990s and early 2000s, CLCuD was managed by planting of resistant cotton varieties. After that, CLCuMuV was rarely detected in a number of field surveys, although a second epidemic of CLCuD occurred in South Asia [19]. However, we found CLCuMuV (Fai[CN:GZ:G6:Hib:06], EF465535) infecting *H. rosa-sinensis* in Guangzhou, China in 2006 [12].

To monitor the spread of CLCuMuV in China, surveys were conducted in the regions shown in Fig. 1 during 2006 – 2014 to document malvaceous plant species, especially *H. rosa-sinensis,* showing typical symptoms associated with begomoviral infection. To confirm infection of CLCuMuV, total DNA was extracted from the infected samples using a CTAB method and used as template for PCR using a degenerate primer pair specific to begomovirus (AV494/CoPR, [8]) and/or a specific primer pair for CLCuMuV. CLCuMuB was detected using β01/β02 [3].

**Fig. 1:**
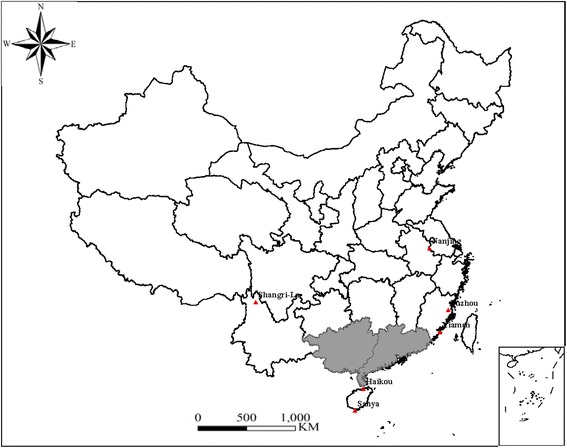
Distribution of Cotton leaf curl Multan virus (CLCuMuV) and its associated betasatellite (CLCuMuB) in China. Guangdong and Guangxi, the two provinces in which CLCuMuV and CLCuMuB has been endemic are shaded. For other provinces, red triangles were used to indicate regions that have been surveyed and CLCuMuV and CLCuMuB have been detected

In 2006, *H. rosa-sinensis* plants showing symptoms typical of begomovirus infection were only identified in Guangzhou, Guangdong, China. The disease was named China rose (*H. rosa-sinensis*) leaf curl disease (CrLCD) according to the symptoms [12]. In the 3 years following thereafter, CrLCD became common in surrounding cities of Guangzhou. In 2008, symptomatic *H. rosa-sinensis* were found in Nanning, Guangxi, China. In 2008 – 2010, CrLCD became established in Guangdong and Guangxi. In some regions of the two provinces, the incidence of the disease has reached 100 %. After 2010, CrLCD began to appear in other provinces of Southern China including Hainan, Fujian, Yunnan, and Jiangsu. Besides *H. rosa-sinensis,* three other malvaceous plant species, namely *H. esculentus*, *Malvaiscus arboreus* and *Gossypium hirsutum* showing similar symptoms with CrLCD were found in Guangdong [7, 23, 24]. In fields growing *H. esculentus* in Guangzhou*,* the incidence of the disease can reach 100 %. In addition, *G. hirsutum* and *H. cannabinus* showing similar symptoms were found in Guangxi and Hainan, respectively ([4] and Our unpublished results). A total of 400 symptomatic samples were collected. Almost all of the samples were found positive for CLCuMuV and CLCuMuB in PCR assays as described above (data not shown).

To gain insights into the population structure of CLCuMuV in China, abutting primer pairs were designed using sequences of the PCR fragments obtained as described above and used to amplify the full genome of representative CLCuMuV isolates. The resulting amplicon was ligated into the vector pMD 20 T (TaKaRa Biotechnology (Dalian) Co., Ltd, Dalian, China). The recombinant plasmid was transformed into *E. coli* DH5α. For each transformation, the inserts of three randomly chosen clones were sequenced by primer walking in both directions. Sequence data were assembled and analyzed using DNAStar (DNAStar Inc., Madison, USA). In all cases, the three clones were more than 99 % identical to each other. Thus, one clone was randomly selected to represent a virus isolate and the sequence was submitted to NCBI. To sequence CLCuMuB, the amplicons derived from the PCR with primers β01/β02 were recovered from the gel and sequenced as described above. A total of 34 CLCuMuV and 30 CLCuMuB isolates were sequenced (all the sequences can be accessed by searching *Cotton leaf curl Multan virus* in Genbank). But in the sequence analysis, 6 CLCuMuV and 6 CLCuMuB sequences reported by other groups (see Additional file 1) were also used. For sequence analysis, MEGA 6.0 was used to align the sequences and visualize sequence variances [22]. DNA polymorphism was analyzed using DnaSP5 (version 5.10.1, http://www.ub.edu/dnasp). SDT.V1 [14] was used to calculate pairwise identities. For all analyses, default parameters of the respective software were adopted.

The nucleotide sequences of the 40 completely sequenced CLCuMuV in China show pairwise identities higher than 99 %. No correlations were found between the extent of divergence and geographical origin, year of collection and host plants of these CLCuMuV isolates. The nucleotide diversity (pi) value is as low as 0.00352. Among the 128 polymorphic (segregating) sites found across the 2739 bp-long genome, 74 % were singleton variable sites. In all cases tested, the CLCuMuV was accompanied by CLCuMuB. The nucleotide sequences of the 36 completely sequenced CLCuMuB show pairwise identities higher than 98 %. Similarly, no correlations were found between the extent of divergence and geographical origin, year of collection and host plants of these CLCuMuB isolates. The pi value is 0.00765. A total of 110 variable (polymorphic) sites were found across the 1326 bp-long genome, with 65 % of them being singleton variable sites.

The CLCuMuV and CLCuMuB found in South Asia show a high genetic diversity [19]. However, all the South Asian isolates of CLCuMuV and CLCuMuB show < 96 % and 92 % nucleotide sequence identities with the Chinese isolates of CLCuMuV and CLCuMuB, respectively. The closest relatives of the Chinese isolates of CLCuMuV in South Asia are isolates belonging to Faisalabad strain of CLCuMuV from Pakistan [19]. Phylogenetic trees were built to clarify relationships between Chinese and South Asian CLCuMuVs. Consistent with the report of Sattar et al. [19], all the isolates of CLCuMuV from China fell within a subclade that clustered with Faisalabad strain of CLCuMuV from Pakistan (Fig. 2).

**Fig. 2:**
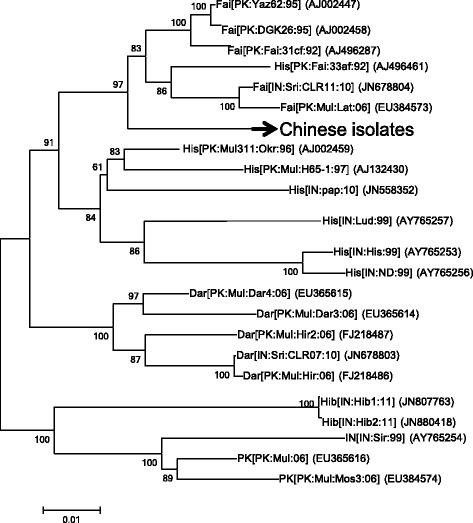
Phylogenetic relationships between Chinese and South Asian CLCuMuVs. Neighbor-Joining analysis was performed with 1000 bootstrap replicates using MEGA6. Branches having branch support value smaller than 50 % were collapsed. For South Asian CLCuMuV isolates, the latest official names were used (talk.ictvonline.org/ictv_wikis/m/files_gemini/5120.aspx). Please see Additional file 1 for GenBank accessions and sequences of Chinese isolates of CLCuMuVs

In all, we report our observation that CLCuMuV invaded Southern China in the last nine years since it was first detected in Guangzhou. We showed that nucleotide sequences of 40 temporally and geographically distinct CLCuMuV isolates infecting different host plants in Southern China show very low genetic variance. We conclude from the high genetic homogeneity of CLCuMuV that a single introduction event is responsible for its introduction into China. Interestingly, high genetic stability was also observed for two other WTGs, *Tomato yellow leaf curl virus*–Mild (TYLCV-Mld[RE]) and *Tomato yellow leaf curl Sardinia virus* (TYLCSV), invading La Reunion island and southern Spain, respectively, after a single introduction event [6, 18].

In the beginning of this century, begomoviruses have emerged as important plant pathogens worldwide. Scientists have begun to pay great attention to this group of viruses [13, 25]. In addition, begomoviruses induce symptoms that are very easy to recognize in malvaceous plant species. Thus, it seems almost unlikely that CLCuMuV had been spread to China long before 2006 without the awareness of local scientists. Likewise, the detection of CLCuMuV in other regions of China was several years later than that in Guangzhou. These strongly suggest a scenario in which CLCuMuV and its associated CLCuMuB first arrived in Guangzhou in 2006 or a few years before. Then, CLCuMuV-CLCuMuB was spread to other regions of China. This is consistent with the fact that Guangzhou is an important port city for international trading and a center of *H. rosa-sinensis* cultivation in China.

The source of the invading virus may be imported *H. rosa-sinensis* plants or viruliferous whiteflies. However, *H. rosa-sinensis* is not an attractive host plant of whiteflies. Additionally, begomoviruses are not transmitted vertically or horizontally by whiteflies. Therefore, if the introduction of CLCuMuV-CLCuMuB is mediated by viruliferous whiteflies, the propagule pressure must be very high. This contradicts with the fact that the incidence of CLCuMuV had been significantly decreased starting from the year 2001 in South Asia [19]. Thus, it may be inferred that imported living *H. rosa-sinensis* is the source of the invading CLCuMuV-CLCuMuB. *H. rosa-sinensis* is a vegetatively propagated perennial shrub widely grown as ornamentals in many regions of Southern China. This means that a single diseased plant is enough for establishment and long-distance dispersal of CLCuMuV-CLCuMuB. Human-mediated dispersal of infected *H. rosa-sinensis* may also be responsible for the high speed with which the virus complex became established in Southern China.

Although closely related, the introduced and known South Asian isolates of CLCuMuV show substantial differences. They share a nucleotide sequence identity < 96 % for CLCuMuV and < 92 % for CLCuMuB. As a diversity center for CLCuMuV, only a small number of CLCuMuV isolates have been completely sequenced in Pakistan. It is possible that the invading CLCuMuV represents an uncharacterized isolate of CLCuMuV occurring in this country, most probably infecting *H. rosa-sinensis*. Further experiments to find this uncharacterized isolate in Pakistan will be useful to clarify the origin of the CLCuMuV invading China.

Five malvaceous plant species, namely *H. rosa-sinensis, H. esculentus*, *Malvaiscus arboreus, G. hirsutum* and *H. cannabinus*, were found to be naturally infected by the invading CLCuMuV in China. All the five plant species have a long cultivation history in South Asia. For *H. esculentus* and *H. cannabinus*, well supported data suggest that the two plant species were introduced from South Asia to China in the recent 100 years [1]. This implies that CLCuMuV is infecting only plants that may have co-evolved with the virus. To put it in another word, the biogeographical homogeneity between the East and South Asia may have facilitated the successful invasion and establishment of CLCuMuV.

As mentioned above, CLCuMuV is a damaging pathogen to cotton production. In this context, it is fortunate that cotton is not widely grown in the regions that have been invaded by CLCuMuV. However, some regions of Southern China are important sites for cotton germplasm preservation. Besides, international trade between China and neighboring Southeast Asian countries has been greatly intensified in recent years due to the establishment of China-ASEAN Free Trade Area (CAFTA). We have found CLCuMuV infecting *H. rosa-sinensis* in some Southeast Asian countries including Laos, Vietnam and the Philippines (Our unpublished results). There are many begomoviruses infecting malvaceous plant species in Southeast Asia. Recombination of CLCuMuV with these viruses may give rise to new virus strains or species that have novel pathogenic properties. Thus, close surveillances of the occurrence and spreading of CLCuMuV in East Asia are needed.

## Additional file

Additional file 1:
**GenBank accessions and sequences of Chinese isolates of CLCuMuVs in this study.** (FAS 111 kb)
